# Establishment and application of a rapid diagnostic method for BVDV and IBRV using recombinase polymerase amplification-lateral flow device

**DOI:** 10.3389/fvets.2024.1360504

**Published:** 2024-03-27

**Authors:** Yan Wang, Jinyuan Shang, Zhijie Li, Ao Zhang, Yuening Cheng

**Affiliations:** Key Laboratory of Economic Animal Diseases, Ministry of Agriculture, Institute of Special Animal and Plant Sciences, Chinese Academy of Agricultural Sciences, Changchun, China

**Keywords:** Bovine Viral Diarrhea Virus, Infectious Bovine Rhinotracheitis Virus, reverse transcriptase recombinase aided amplification, lateral flow dipstick, rapid detection

## Abstract

Bovine Viral Diarrhea Virus (BVDV) and Infectious Bovine Rhinotracheitis Virus (IBRV) are the two most prevalent infectious diseases in cattle. They both can cause persistent infection and immunosuppression, resulting in significant economic losses in the livestock industry. Therefore, rapid detection of early BVDV and IBRV infections is crucial. In this study, a method for the rapid detection of BVDV and IBRV was established by using recombinase polymerase amplification (RPA) combined with lateral flow device (LFD). By optimizing the temperature and time conditions of the RPA reaction, the sensitivity, specificity, and clinical performance were evaluated. The results indicated that the RPA reaction could be completed at 40°C within 25 min. The LOD for BVDV and IBRV by RPA-LFD were 5.1 × 10^1^ copies/μL and 6.65 × 10^1^ copies/μL, respectively, with no cross-reactivity observed with other viruses such as CSFV, BRSV, BPIV3, BRV, and BCoV. Testing of 32 clinical samples showed consistent results between RPA-LFD and qPCR. The RPA-LFD method established in this study can be used for the rapid clinical detection of BVDV and IBRV, which providing a rapid and convenient molecular biology approach for on-site rapid detection and epidemiological investigations. Simultaneously, it offers technical support for the prevention and control of these viruses.

## 1 Introduction

Bovine Viral Diarrhea Virus (BVDV), belonging to the *Pestivirus* genus within the Flaviviridae family, is an enveloped, single-stranded positive-sense RNA virus (1). Its genome encompasses crucial structural protein genes, including p14 (encoding the core protein), gp48 (encoding the membrane protein E^rns^), gp25 (encoding the membrane protein E1), and gp53 (encoding the membrane protein E2) (2). Additional non-structural protein genes, namely E^rns^, E1, and E2, contribute to the composition of the virus structural proteins situated on the virus surface. Erns is a highly conserved and neutralizing epitope-containing protein in the BVDV-encoded proteins, making it a popular structural protein for BVDV gene engineering vaccines and diagnostic reagents (3). Considering Erns high conservation, even at low virus concentrations, it is a suitable detection antigen (4) making it crucial for BVDV detection (5). Therefore, this study focuses on the conserved region of Erns for subsequent experiments. BVDV exhibits a broad host range, infecting various species such as cattle, camels, pigs, sheep, and deer (6). The virus induces an acute, febrile, and highly contagious infectious disease in animals. Clinically, it manifests with symptoms such as severe diarrhea, abortion in pregnant cows, reproductive disorders, and immune dysfunction, contributing to the overall severity of the condition (7). Some studies have shown that the current BVDV infection rate in Chinese herds is around 36.0%, with the highest BVDV antigen-positive rate in samples from the northwestern region (8). The disease primarily impacts cattle, displaying susceptibility across all age groups, with calves exhibiting the highest susceptibility. Prolonged viral shedding from persistently infected calves contributes to extensive disease spread within cattle herds, resulting in significant economic losses in the livestock industry (9). Cattle persistently infected with BVDV must be isolated because of their prolonged viral shedding, contributing to the spread of the disease.

Infectious Bovine Rhinotracheitis (IBR): IBR, caused by the Infectious Bovine Rhinotracheitis Virus (IBRV), is an acute, contact-transmissible infectious disease. IBRV belongs to the Varicellovirus genus within the Alphaherpesvirinae subfamily of the Herpesviridae family, characterized as a double-stranded enveloped DNA virus (10). gG is one of the main envelope glycoproteins of IBRV, with strong antigenicity and conservation (11). IBRV commonly causes respiratory infections in cattle, displaying features of fever, acuteness, and contact transmission. Clinical symptoms primarily include respiratory manifestations such as rhinitis, nasal discharge, breathing difficulties, and fever (12). Additionally, IBR can induce reproductive tract infections, conjunctivitis, abortion, mastitis, and infections of the nervous system in newborn calves, potentially triggering bovine encephalitis under specific conditions (13). The disease can cause immunosuppression, leading to mixed infections with various pathogens, posing a severe threat to the cattle industry in China. Hence, there is an urgent need to establish rapid and effective diagnostic methods for the prevention and control of the rapid progression of the disease (14). Accurate and timely detection of BVDV and IBRV is crucial for early diagnosis, prevention, and control of this disease. However, the limitations of current detection methods highlight the urgent need for a rapid and reliable approach. Early identification of persistently infected cattle is essential for effective disease management.

Currently, prevalent diagnostic approaches encompass clinical diagnosis, serological diagnosis, and molecular biology techniques. Molecular methods commonly utilized include reverse transcription PCR, nested PCR, real-time quantitative fluorescent PCR, and Loop-Mediated Isothermal Amplification (LAMP) (15). While these techniques boast high accuracy, they often prove time-consuming, intricate in operation, and demand sophisticated equipment, requiring high cost of the equipment and trained technicians. Consequently, they are not easily adaptable to rudimentary laboratory settings, which is unfavorable for extensive clinical diagnostic testing. Hence, there is an urgent need to establish an efficient, sensitive, and accurate dual detection method to meet the demands of extensive clinical diagnosis for BVDV and IBRV. Recombinase Polymerase Amplification (RPA) technology is an isothermal amplification technique that mimics the replication mechanism of DNA and relies on recombinase, single-stranded binding proteins (SSB), and DNA polymerase for amplification. Recombinase binds to primers to form a protein-DNA complex, which searches for homologous sequences in the DNA, and then undergoes a strand exchange reaction with strand-replacing DNA polymerase to begin the amplification of the DNA, and the replaced DNA binds to the SSB, which prevents further replacement (16). Following amplification, result can be observed using lateral flow Device (colloidal gold test strips), offering the advantage of lower equipment requirements and rapid testing even in basic laboratories. This study aims to establish a dual detection method for BVDV and IBRV using the RPA-LFD approach, with validation through clinical sample testing. This innovative detection method can provide a practical, sensitive, and accurate technical reference for rural veterinary laboratories.

## 2 Methods and materials

### 2.1 Pathogens, clinical samples, and DNA/RNA extraction

IBRV, BVDV, Classical Swine Fever Virus (CSFV), Bovine Respiratory Syncytial Virus (BRSV), Bovine Parainfluenza Virus 3 (BPIV3), Bovine Rotavirus (BRV), and Bovine Coronavirus (BCoV) were isolated and preserved in the laboratory. Thirty-two clinical serum samples were collected from diseased cattle in different areas of Jilin Province and stored at −80°C. Following the instructions, viral RNA was extracted by using Trizol reagent (Solarbio, China), and subsequently reverse transcribed into cDNA using the TransGen Biotech reverse transcription kit (China). The separation of IBRV DNA was performed using the EasyPure^®^ Viral DNA Kit (TransGen Biotech, China). All cDNA and DNA samples were then stored at −80°C.

### 2.2 Primer and probe design and synthesis

Sequences of different subtypes of BVDV and IBRV were downloaded from the NCBI GenBank database (http://www.ncbi.nlm.nih.gov). Using MEGA 7.0 software, sequence alignments were performed for different strains. Based on the conserved regions of the E^rns^ gene of the BVDV strain BVDVJL-1 (GenBank: KF501393.1) and the gG gene of the IBRV strain (GenBank: MK552112.1), four pairs of specific primers and probes were designed using Oligo7.0 software (Table 1). The 5′ end of the reverse primers for BVDV was labeled with biotin. And the 5′ end of the reverse primers for IBRV was labeled with Dig. The probe 5′ end modification was labeled with 6-carboxyfluorescein (FAM), connected to the adjacent downstream oligonucleotide through a dSpacer, and had a C3 spacer at its 3′ end. All primers and probes were synthesized by Sangon Biotech Co., Ltd (Shanghai).

**Table 1:** Primer and probe sequences for BVDV and IBRV.

**Primers and probes**	**Sequence (5^′^ → 3^′^)**	**Amplification size (bp)**	**Modification**
BVDV-F	GGATCC-CTTCCCATCTAGCCACCGATATGG	554	
BVDV-R	AAGCTT-CGTATGCTCCAAACCACGTCTTAC		
BVDV-RPA-F	CAC AAG CTA GAG ATA GCC CCA CAC TCT TGA CA	296	
BVDV-RPA-R	CTC CAA ACC ACG TCT TAC TCT TGT TTT CCA ACT		5′ Biotin
BVDV-RPA-P	TGA CGG GAT GAC CAA CTC CTT AGA AAA TGC-dspacer-CAG ACA AGGAAC CGC TA		5′ 6-FAM, dSpacer; 3′ C3 Spacer
q-E^rns^-F	CATTACCTTGTTGACGGGATG	99	
q-E^rns^-R	TCCCTAGTATCCCGAGCTG		
IBRV-F	GGATCC-GCCTCGCGTACCTGCAC	608	
IBRV-R	AAGCTT-CTACCCCGAGGCCGC		
IBRV-RPA-F	CTG GCC GTG CAC GCC GAA GAG TTT CCA CCC	282	
IBRV-RPA-R	TAC CCC GAG GCC GCA CCG AAG ACG GGG TGC TC		5′ Dig
IBRV-RPA-P	GAG CAC GCG CCG AGA GCC GCA CAG CCC GTA -dspacer-CCG CTG CCT4GGC GGT G		5′ 6-FAM, dSpacer; 3′ C3 Spacer
q-IBRV-F	CCGAGAGCCGCACAGCCCGTA	152	
q-IBRV-R	CCCGAGGCCGCACCGAAGAC		

F, forward primer; R, reverse primer; P, probe; Dig, Digoxigenin; FAM, Carboxy fluorescein; dSpacer A tetrahydrofuran residue; C3-spacer 3′ -block.

### 2.3 Construction of positive plasmids

Using cDNA or DNA from BVDV and IBRV as templates, PCR amplification was performed with specific primers. The purified PCR products were then separately ligated with pBlueScript KS II using BamHI and HindIII restriction endonucleases. The resulting recombinant plasmids were then 10-fold diluted, with the concentration range for BVDV plasmids being 5 × 10^7^ to 5 × 10^1^ copies/μL, and for IBRV plasmids being 6 × 10^7^ to 5 × 10^1^ copies/μL.

### 2.4 Establishment of the RPA-LFD reaction system

**Figure 1:**
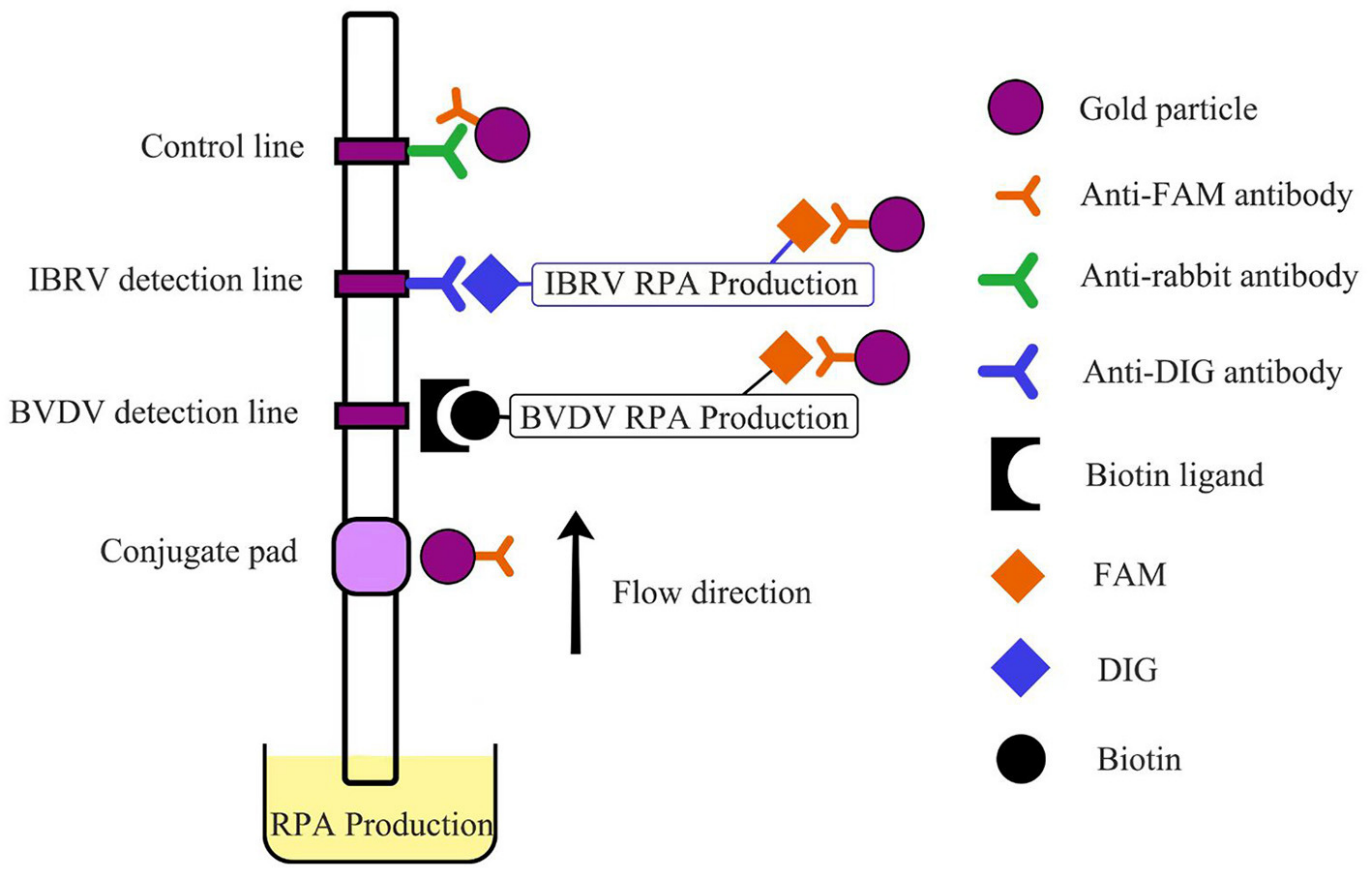
Schematic representation of LFD.

Following the instructions of the TwistAmp^®^ nfo (TwistDx, UK), multiple reaction systems were prepared in 1.5 mL centrifuge tubes, with a total reaction volume of 50 μL. The composition of the reaction system was as follows: Rehydration Buffer 29.5 μL, 10 μmol/L forward and reverse primers each 2.1 μL, probe 0.6 μL, standard positive plasmid template 5 μL, ddH_2_O 8.2 μL. After pre-mixing, the mixture was evenly added to 8-tube strips containing lyophilized enzyme powder. Finally, 2.5 μL of magnesium acetate was added to the tube cap, centrifuged, and the mixture was rapidly mixed for the initiation of the amplification reaction. The reaction mixture was then placed in a water bath for incubation. Carefully opening the lid, 20 μL of the reaction mixture was mixed with 80 μL of detection buffer. The study utilized the Hybridetect 2T (Milenia Biotec GmbH, Germany) test strip, employing gold particle lateral flow technology. This type of test strip is designed for the creation of a qualitative rapid detection system. Following the RPA reaction, the test strip is immersed in the RPA production. The initially FAM and biotin-labeled BVDV complex binds to the gold-labeled FAM-specific antibody (from rabbit) in the conjugate pad of the strip. Simultaneously, the FAM and DIG-labeled IBRV complex also binds to this area. Capillary forces facilitate the diffusion of gold complexes on the analytical membrane. Only the captured RPA production gold particles bind to the respective detection lines (BVDV detection line–BVDV RPA production–gold particles, IBRV detection line–IBRV RPA production–gold particles) when they surpass the immobilized biotin ligand molecules or anti-DIG antibody, producing a visible red-blue band over time. Unbound gold particles flow past the control band and are captured by anti-rabbit antibodies (Figure 1).

### 2.5 Optimization of RPA-LFD reaction conditions

To enhance the amplification efficiency of BVDV and IBRV, optimization was conducted for the reaction temperature (25°C, 30°C, 35°C, 40°C, 45°C, 50°C) and reaction time (5 min, 10 min, 15 min, 20 min, 25 min, 30 min, 35 min). The optimal conditions for the RPA-LFD reaction for detecting both viruses were established based on the detection results.

### 2.6 Sensitivity and specificity analysis

The constructed positive plasmids for BVDV and IBRV were diluted tenfold. The dilution range for BVDV was 5.1 × 10^7^ to 5 × 10^1^ copies/μL, and for IBRV, it was 6.65 × 10^7^ to 6 × 10^1^ copies/μL. The Limit of Detection (LOD) was determined based on the naked eye's ability to clearly observe the detection lines. To assess the specificity of the method, the optimized RPA-LFD detection method established in this study was employed to test clinically similar viruses, including Classical Swine Fever Virus (CSFV), Bovine Respiratory Syncytial Virus (BRSV), Bovine Parainfluenza Virus 3 (BPIV3), Bovine Rotavirus (BRV), and Bovine Coronavirus (BCoV). Distilled water served as the negative control, and the results were observed after the reaction.

### 2.7 Clinical sample detection

To validate the detection effectiveness of RPA-LFD in practical applications, 32 bovine serum samples from cattle showing clinical symptoms were simultaneously tested using RPA-LFD and quantitative Polymerase Chain Reaction (qPCR), and the results of these two methods were compared.

### 2.8 Real-time qPCR

BVDVused two-step qPCR, IBRV used one-step qPCR. The optimized reaction system (20 μL) was determined as follows: template 1 μL, 10 μmol/L forward and reverse primers each 0.5 μL, 2 × RealStar Green Fast Mixture (GenStar, China) 10 μL, ddH_2_O 8 μL. Both DNA and cDNA used an amplification program consisted of 2 min at 95°C, followed by 40 cycles of 15 s at 95°C and 20 s at 60°C. All samples were run in triplicate.

## 3 Results

### 3.1 Optimal reaction temperature and time

**Figure 2:**
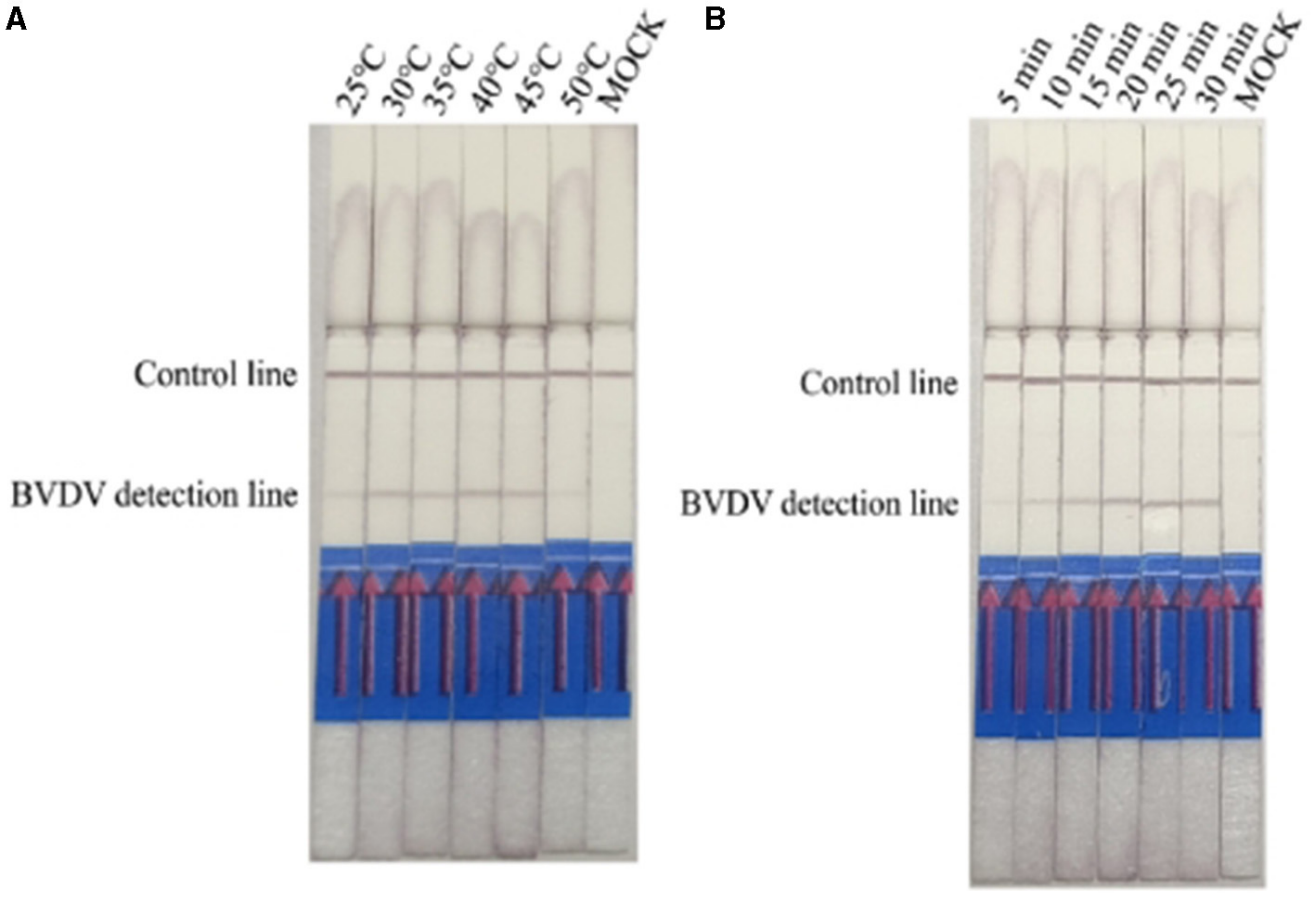
Optimizing detection conditions in the RPA-LFD assay. **(A)** The reaction temperature of BVDV-RPA ranged from 25°C to 50 °C. **(B)** The reaction time of BVDV-RPA ranged from 5 min to 30 min.

The reaction temperature and time were optimized based on the reaction system prepared with the RPA-LFD kit. Using the established RPA-LFD system with recombinant plasmids as templates, reactions were conducted at different temperatures to determine the optimal reaction temperature. The time of exposure for assessment ranged from 5 to 10 min. The results indicated that BVDV amplification products could be detected at reaction temperatures ranging from 25°C to 45°C, with the best detection observed at an incubation temperature of 40°C (Figure 2A). At a reaction temperature of 40°C, the color of LFD bands gradually deepened with increasing time, and the optimal reaction time was between 20 min and 30 min (Figure 2B).

**Figure 3:**
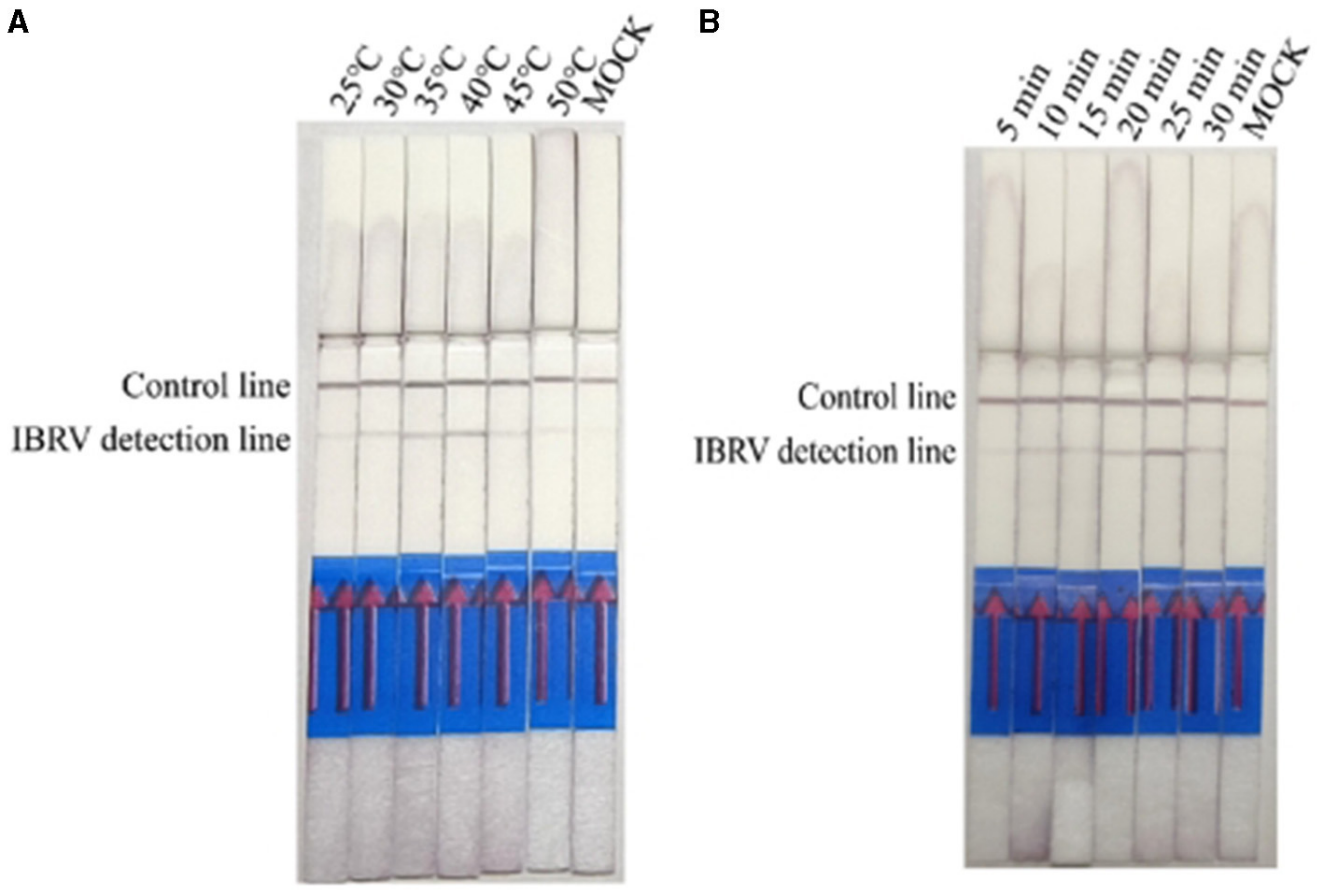
Optimizing detection conditions in the RPA-LFD assay. **(A)** The reaction temperature of IBRV-RPA ranged from 25 °C to 50 °C. **(B)** The reaction time of IBRV-RPA ranged from 5 min to 30 min.

Results of IBRV indicate that IBRV amplification products can be detected at temperatures ranging from 25°C to 45°C, with the optimal detection observed at an incubation temperature of 40°C (Figure 3A). At a reaction temperature of 40°C the color of the detection line on the lateral flow dipstick (LFD) deepened gradually with increasing time, and the best detection effect was achieved at 25 min (Figure 3B). Since mixed infections of Bovine Viral Diarrhea Virus (BVDV) and Bovine Infectious Rhinotracheitis Virus (IBRV) are common in clinical cases, establishing a method for simultaneous detection of both viruses using RPA-LFD enables rapid and efficient differentiation and diagnosis. Therefore, this study selected a temperature of 40°C and a time of 25 min as the optimal reaction conditions for RPA-LFD detection of these two viruses.

### 3.2 Sensitivity and specificity analysis

**Figure 4:**
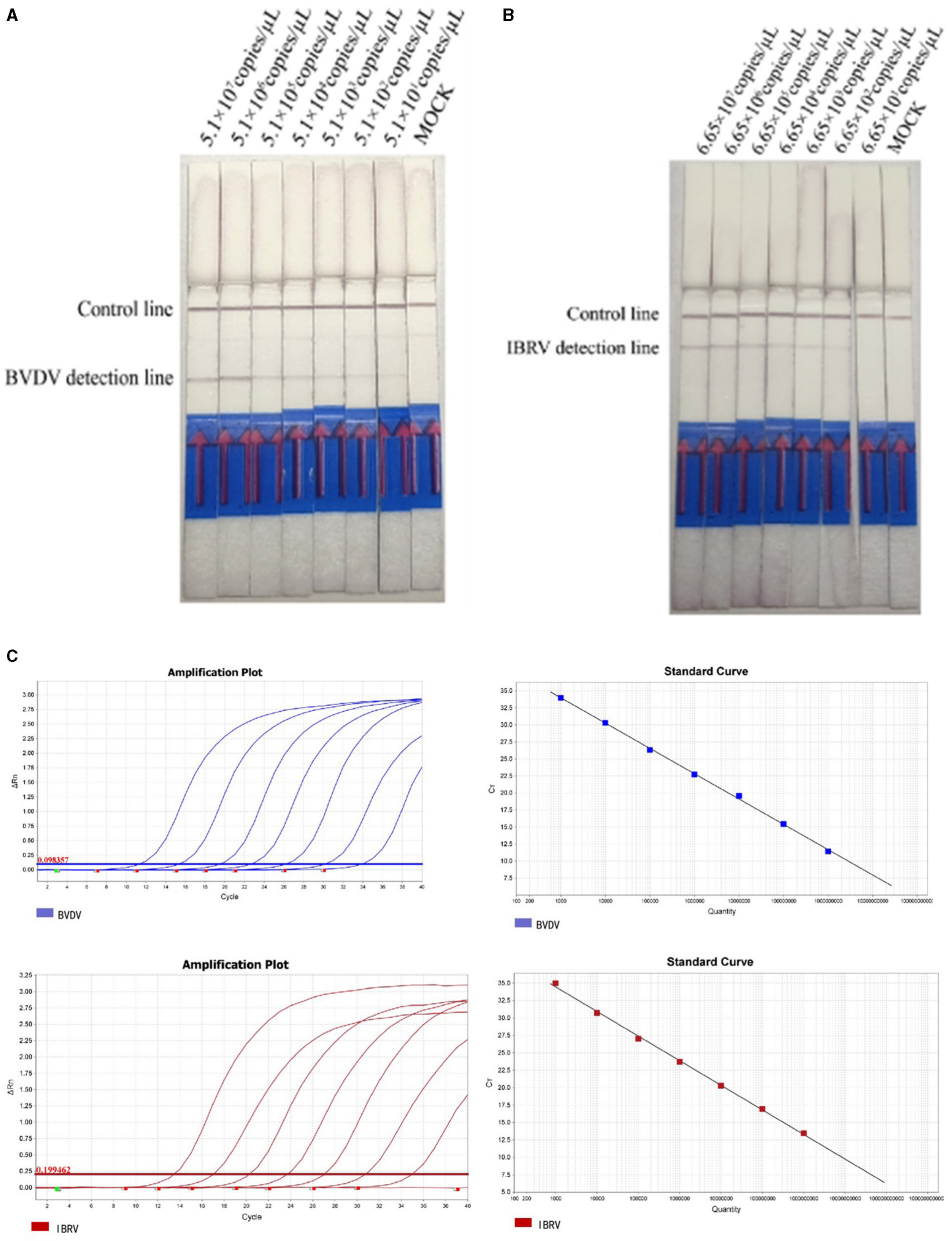
Analyzing the duplex RPA-LFD method sensitivity. **(A)** The dual RPA-LFD detection system can detect 51.0 copies/reaction standards BVDV plasmids. **(B)** The LOD of the method was 66.5 copies IBRV plasmids per reaction. **(C)** The sensitivities and standard curve of RealStar Green Fast Mixture real-time PCR established for the 10-fold serial dilutions of BVDV and IBRV standard RNA. Lanes 1–7: 10^7^, 10^6^, 10^5^, 10^4^, 10^3^, 10^2^, and 10^1^ copies of the template, 8: Mock.

To ensure the accuracy of dilution, positive plasmids were diluted and detected using both qPCR and RPA-LFD. The sensitivity results of the RPA-LFD method indicated that the LOD for BVDV and IBRV were 5.1 × 10^1^ copies/μL and 6.65 × 10^1^ copies/μL, respectively (Figure 4). The qPCR Cycle Threshold (Ct) values were 30.2 and 30.7. The amplification efficiencies for the qPCR assays of BVDV and IBRV are 91.5% and 93.3%, respectively. The detection limits of RPA LFD were consistent with qPCR.

**Figure 5:**
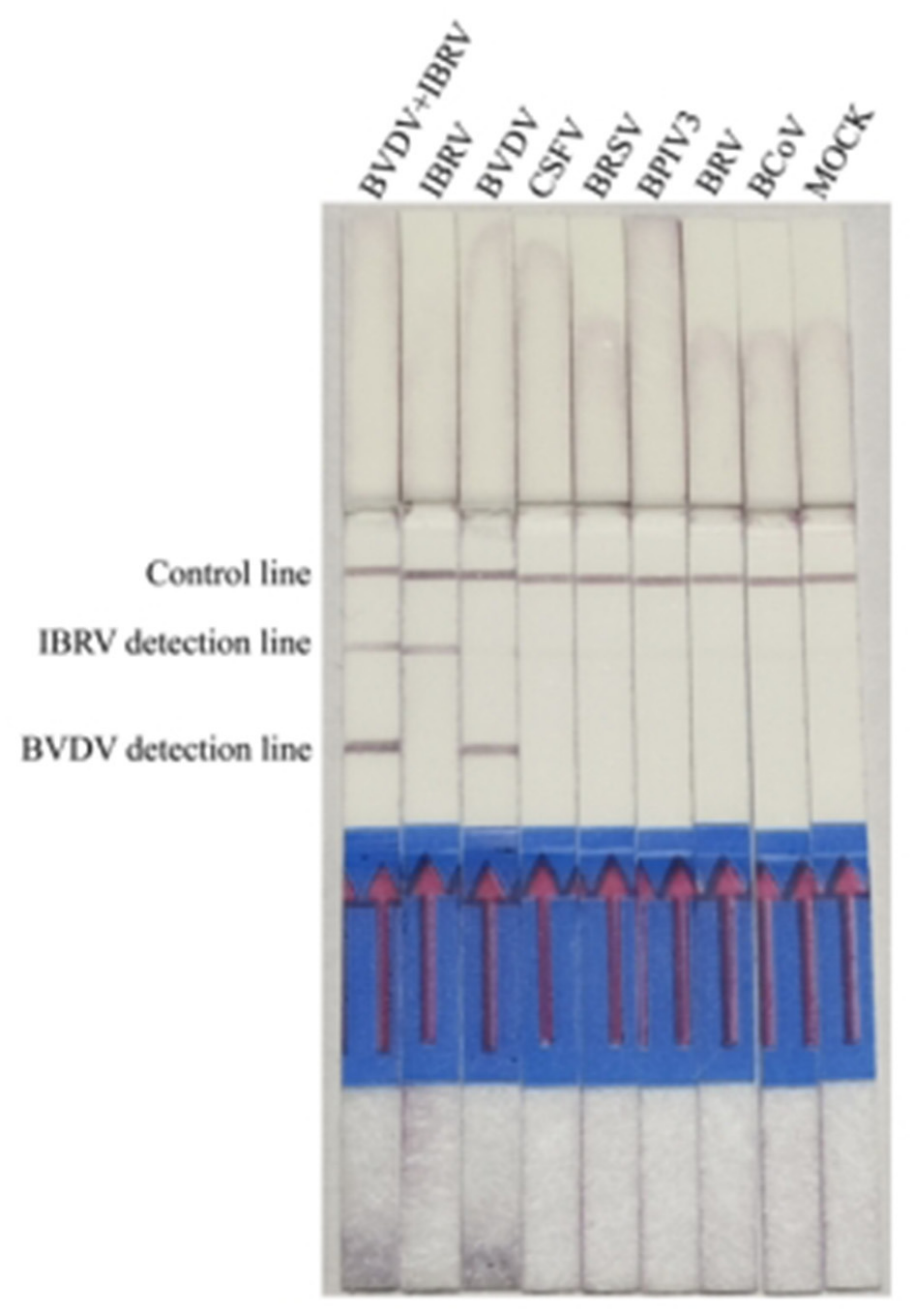
Analyzing the duplex RPA-LFD method specificity. Lanes 1 to 8: BVDV and IBRV, IBRV, BVDV, CSFV, BRSV, BPIV3, BRV, and BCoV; Lane 8: Mock.

Under the optimized reaction conditions, the specificity of the Recombinase Polymerase Amplification with Lateral Flow Dipstick (RPA-LFD) detection method was assessed for seven viruses, including BVDV, IBRV, CSFV, BRSV, BPIV3, BRV, and BCoV. The results indicated that RT-RPA LFD simultaneously detected BVDV and IBRV as positive, while other samples were negative, and there was no cross-reactivity with other viruses (Figure 5).

### 3.3 Clinical sample detection

**Figure 6:**
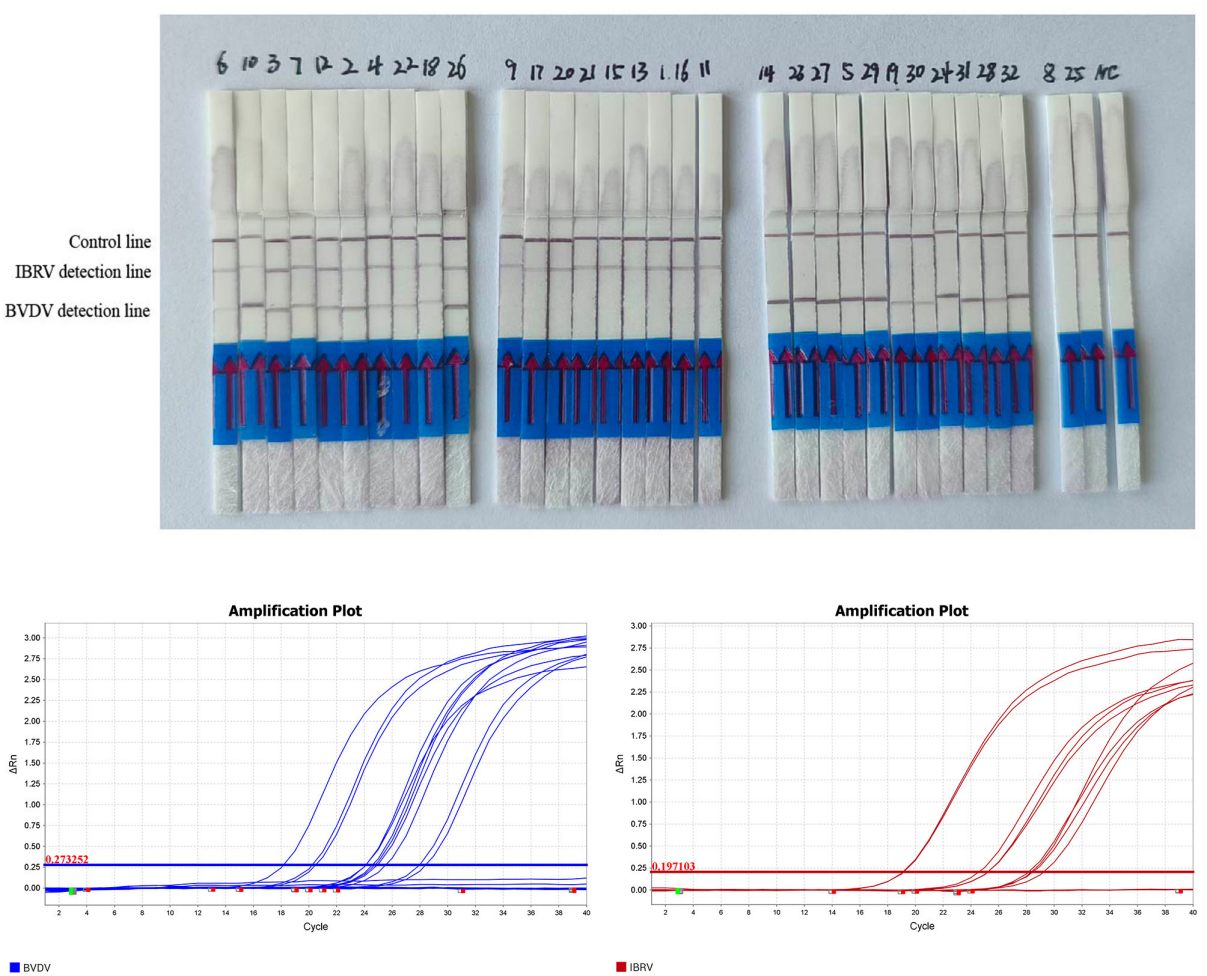
RPA-LFD detection results for 32 clinical samples. Results of qPCR detection for 32 clinical samples of BVDV and IRBV.

Thirty-two clinical samples were simultaneously tested using both RPA LFD and qPCR methods. The results showed that both methods produced consistent results: 21 samples were positive for BVDV and 11 were negative, with a positivity rate of 65%; for IBRV, 19 samples were positive and 13 were negative, with a positivity rate of 59%; It involves a mixed infection of 10 samples. All positive results from the tests (Table 2, Figure 6) were consistent with qPCR. Compared to qPCR, RPA-LFD demonstrated a shorter detection time, more convenient operation, and greater suitability for on-site rapid testing.

**Table 2:** Analysis of detection results for clinical samples by RPA-LFD and qPCR.

**Method**	**Number of samples**	**BVDV**		**IBRV**		**BVDV+IBRV**
		**Positive**	**Negative**	**Positive**	**Negative**	**Positive**
RPA LFD	32	21	11	19	13	10
qPCR	32	21	11	19	13	10

## 4 Discussion

Bovine Viral Diarrhea Virus (BVDV) and Infectious Bovine Rhinotracheitis Virus (IBRV) are the two most common infectious diseases in cattle. The BVDV virus, upon entering the body, causes persistent infection and immune suppression, leading to the invasion of other viruses. Therefore, rapid diagnosis of early BVDV and IBRV infections is crucial. There is an urgent need to develop a detection method for mixed infections of these related pathogens.

Some studies suggest the effective detection of E. miricola using RPA-LFD and exo RPA at 38°C within 30 min, achieving a sensitivity of 102 copies/μL, which is 10-fold higher than generic PCR assays (17). In another study focused on LYCIV detection, various methods were developed, including RPA, real-time RPA, and RPA-conjugate lateral flow dot detection (RPA-LFD). Both RPA and real-time RPA demonstrated the capability to detect viral DNA down to 102 copies/μLL, while RPA-LFD had a slightly lower limit of detection at 101 copies/μL comparable to that of RPA-LFD. Importantly, RPA-LFD showed no cross-reactivity with other waterborne pathogens (18). Similarly, for the visual detection of Porcine Epidemic Diarrhea Virus (PEDV), a reverse transcription (RT)-RPA assay combined with lateral flow dipstrip (LFD) was established, targeting the N gene. The RT-RPA-LFD assay detected as low as 102 copies/μL of PEDV genomic RNA standard, demonstrating high specificity without cross-reactivity with common swine pathogens (19).

This study has established an RPA-LFD method for checking BVDV, with a reaction completed after 25 min of incubation at 35°C. The method's minimum detection limit is 60 copies/μL. Specificity analysis indicates no cross-reactivity with other viruses, and clinical sample testing is consistent with RT-qPCR results (20). Studies have shown that on-site rapid BVDV detection using the RPA-LFD method can be completed within 30 min, offering advantages such as high sensitivity, strong specificity, simple reaction procedures, and short detection times. This method is suitable for quick, accurate, and convenient BVDV testing in cattle farms (21).

In this study, the BVDV and IBRV RPA-LFD method was established, demonstrating high sensitivity with LOD of 5.1 × 10^1^ copies/μL and 6.65 × 10^1^ copies/μL, respectively. In clinical sample testing, the positive detection rates for BVDV and IBRV were 65% and 59%, respectively, and the positive detection rate for mixed infections of the two viruses was 31%. These results are consistent with those obtained using qPCR detection methods. Considering the high prevalence of BVDV in breeding farms, followed by IBRV, and the occurrence of mixed infections of BVDV and IBRV, the RPA-LFD technique established in this study provides a highly sensitive and widely applicable method for rapid detection. The results of clinical sample testing show that the RPA-LFD method is consistent with the qPCR method. Therefore, the RPA-LFD method established in this study can be used for rapid clinical detection of BVDV and IBRV, providing a fast and simple molecular biology method for the diagnosis and epidemiological investigation of BVDV and IBRV in cattle herds in China, while also supporting their prevention and control.

Emphasizing the significance of conducting a thorough validation of the assay across multiple laboratories to establish its robustness and consistency, given the pivotal role this experiment plays in shaping our future research. As we progress, our focus will extend beyond optimization, encompassing an in-depth exploration of potential clinical applications in diverse contexts. Additionally, we plan to expand our investigations to include larger and more varied sample sets, enhancing the depth and reliability of our findings for subsequent research initiatives.

## Data availability statement

The original contributions presented in the study are included in the article/supplementary material, further inquiries can be directed to the corresponding author.

## Ethics statement

The animal study was approved by Institute of Special Animal and Plant Sciences, Chinese Academy of Agricultural Sciences. The study was conducted in accordance with the local legislation and institutional requirements.

## Author contributions

YW: Writing – original draft, Writing – review & editing. JS: Data curation, Formal analysis, Writing – original draft, Writing – review & editing. ZL: Conceptualization, Methodology, Writing – original draft, Writing – review & editing. AZ: Software, Validation, Writing – original draft, Writing – review & editing. YC: Funding acquisition, Resources, Writing – original draft, Writing – review & editing.
